# Primate-specific isoform of Nedd4-1 regulates substrate binding via Ser/Thr phosphorylation and 14-3-3 binding

**DOI:** 10.1038/s41598-023-44761-9

**Published:** 2023-10-20

**Authors:** George Kefalas, Daniela Rotin

**Affiliations:** 1 Cell Biology Program, the Hospital for Sick Children, PGCRL 19-9715, 686 Bay Street, Toronto, ON M5G 0A4 Canada; 2 Biochemistry Department, University of Toronto, Toronto, ON M5G 0A4 Canada

**Keywords:** Ubiquitin ligases, Biochemistry, Proteomics

## Abstract

Nedd4 (Nedd4-1) is an E3 ubiquitin ligase involved in crucial biological processes such as growth factor receptor signaling. While canonical Nedd4-1 comprises a C2-WW_(4)_-HECT domain architecture, alternative splicing produces non-canonical isoforms that are poorly characterized. Here we characterized Nedd4-1(NE), a primate-specific isoform of Nedd4-1 that contains a large N-terminal Extension (NE) that replaces most of the C2 domain. We show that Nedd4-1(NE) mRNA is ubiquitously expressed in human tissues and cell lines. Moreover, we found that Nedd4-1(NE) is more active than the canonical Nedd4-1 isoform, likely due to the absence of a C2 domain-mediated autoinhibitory mechanism. Additionally, we identified two Thr/Ser phosphoresidues in the NE region that act as binding sites for 14-3-3 proteins, and show that phosphorylation on these sites reduces substrate binding. Finally, we show that the NE region can act as a binding site for the RPB2 subunit of RNA polymerase II, a unique substrate of Nedd4-1(NE) but not the canonical Nedd4-1. Taken together, our results demonstrate that alternative splicing of the ubiquitin ligase Nedd4-1 can produce isoforms that differ in their catalytic activity, binding partners and substrates, and mechanisms of regulation.

## Introduction

Ubiquitination is a post-translational modification that leads to a variety of outcomes, primarily targeting proteins for degradation, as well as other non-proteolytic functions. This process involves the coordinated action of E1, E2 and E3 (ubiquitin ligase) enzymes^1^. The E3 is responsible for recognizing substrates and catalyzing the covalent attachment of ubiquitin onto them. The human genome encodes hundreds of E3 ubiquitin ligases, nine of which belong to the Nedd4 (neuronal precursor cell-expressed developmentally downregulated 4) family^2^. Nedd4-1, also known as Nedd4, is the prototypic member of the Nedd4 family. Various roles for Nedd4-1 have been reported, most notably in the regulation of growth factor receptor signaling^3–5^. Accordingly, Nedd4-1 has been implicated in physiological processes such as animal growth and pathological conditions such as cancer^6,7^.

Members of the Nedd4 family typically contain an N-terminal C2 domain involved in subcellular localization^8^ and autoinhibition of catalytic activity^9–11^, two to four central WW domains that usually mediate substrate recognition^12,13^, and a C-terminal catalytic HECT domain^14^. However, alternative splicing has been reported to produce non-canonical isoforms of multiple Nedd4 family members that do not adhere to this common domain architecture^15–17^. Alternatively spliced isoforms of human Nedd4-1 have been identified in cDNA libraries^18^, but their function and significance are unknown. Here we describe a primate-specific Nedd4-1 isoform bearing an N-terminal extension (NE) and analyzed the expression and activity of this isoform. Furthermore, we identified an isoform-specific regulatory mechanism of substrate binding mediated by 14-3-3-interacting phosphosites in the NE region.

## Results

### Alternative splicing produces an N-terminally extended isoform of Nedd4-1

**Figure 1:**
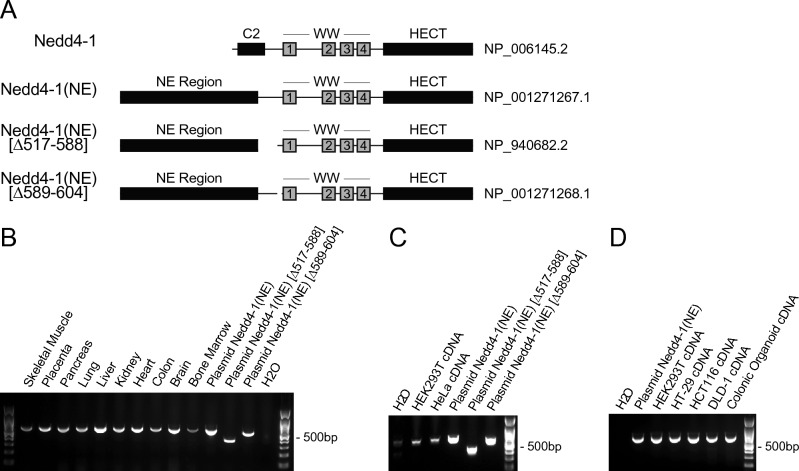
Alternative splicing of Nedd4-1(NE). (**A**) Schematic representation of human Nedd4-1 isoforms formed by alternative splicing. Protein accession numbers (NCBI) are listed on the right of each isoform. Nedd4-1, the canonical isoform, contains a C2 domain, 4 WW domains, and a HECT domain. Due to alternative splicing, Nedd4-1(NE) contains a 516-amino acid NE region and a partial C2 domain. Two other isoforms, Nedd4-1(NE) [Δ517-588] and Nedd4-1(NE) [Δ589-604], contain the same NE region, with amino acids missing downstream of the NE region as indicated in their names. Nedd4-1(NE) mRNA expression in (**B**) human tissues, or (**C**,**D**) human cell lines and colonic organoids, was assessed by PCR using primers that can recognize all three alternatively spliced isoforms of Nedd4-1(NE) and generate amplicons of different sizes (predicted amplicon lengths: Nedd4-1(NE) 613bp; Δ517-588 397bp; Δ589-604 565bp). Plasmids were used as positive controls.

The canonical human Nedd4-1, which comprises a C2-WW_(4)_-HECT domain architecture (Fig. 1A), has been well characterized in many studies. However, other alternatively spliced isoforms of human Nedd4-1 are poorly characterized, including at least three isoforms that contain a common 516-amino acid NE region which replaces most of the C2 domain (Fig. 1A, Fig. S1A). The NE region does not contain any known domains or predicted structures and is not found in other Nedd4 family members. While Nedd4-1, especially the HECT domain, is well conserved throughout evolution, the NE region is only highly conserved amongst higher order mammals such as primates (Fig. S1B). Given the presence of this NE region, we have named the longest of these isoforms Nedd4-1(NE).

To confirm the expression of these alternatively spliced isoforms of human Nedd4-1, we used a cDNA panel derived from mRNA extracted from various human tissues. We confirmed mRNA expression of Nedd4-1(NE), but not Nedd4-1(NE) [Δ517-588] or Nedd4-1(NE) [Δ589-604] isoforms, across all tissues tested (Fig. 1B). Moreover, we found the same pattern of isoform expression across various human cell lines and colonic organoids (Fig. 1C,D). Together, these data suggest that alternative splicing produces ubiquitous expression of the Nedd4-1(NE) isoform in humans. Although Nedd4-1(NE) mRNA is widely expressed in human cells, its expression levels relative to the canonical Nedd4-1 are significantly lower (Fig. S2), and accordingly, we were unable to detect endogenous protein expression of this isoform using antibodies specific to the NE region that we generated. This could be due to the low abundance of the Nedd4-1(NE) protein in these cells, at least under steady-state conditions, low sensitivity of the antibodies, or both.

### Nedd4-1(NE) exhibits enhanced catalytic activity compared to Nedd4-1

**Figure 2:**
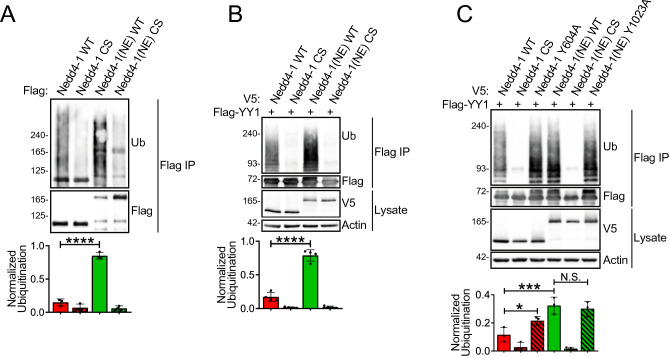
Nedd4-1(NE) displays enhanced ubiquitin ligase activity compared to Nedd4-1. HEK293T cells were transfected with the indicated constructs. Cell lysates were subjected to Flag IP following addition of 1% SDS and boiling to dissociate protein complexes. Ubiquitination (Ub) of (**A**) 3xFlag-tagged Nedd4-1 isoforms, or (**B**,**C**) Flag-YY1 was detected by immunoblotting. Catalytically inactive (Cys → Ser; CS) mutants were used as negative controls. Data are means ± S.D. of 3–4 independent experiments. Statistical significance was determined using (**A**,**B**) Student’s t test, or (**C**) one-way ANOVA (*p < 0.05; ***p < 0.001; ****p < 0.0001; *N.S.* not significant).

To better understand the role of the NE region in Nedd4-1(NE) function, we first compared the catalytic (ubiquitin ligase) activity of Nedd4-1(NE) with the canonical isoform of Nedd4-1. To do so, we transfected HEK293T cells with N-terminally Flag-tagged Nedd4-1 or Nedd4-1(NE) and assessed their ability to catalyze the transfer of ubiquitin onto themselves (i.e., autoubiquitination). Lysates from transfected cells were boiled in SDS to remove Nedd4-1- or Nedd4-1(NE)-interacting proteins, and autoubiquitination was determined by immunoprecipitating (IP) with anti-Flag antibodies and immunoblotting with anti-ubiquitin (Ub) antibodies. Interestingly, Nedd4-1(NE) was autoubiquitinated at higher levels than Nedd4-1 (Fig. 2A). To study catalytic activity towards a cellular substrate, we analyzed the ability of Nedd4-1 and Nedd4-1(NE) to catalyze the transfer of ubiquitin onto the transcription factor YY1, a known substrate of Nedd4-1, which is ubiquitinated by Nedd4-1 without affecting its (YY1) stability^19^. Consistent with the autoubiquitination data, we found that Nedd4-1(NE) was able to ubiquitinate YY1 to a greater extent than Nedd4-1 (Fig. 2B). Together, these data indicate that Nedd4-1(NE) displays enhanced ubiquitin ligase activity compared to canonical Nedd4-1.

Next, we sought to understand the basis behind the increased catalytic activity of Nedd4-1(NE). In the case of Nedd4-1, an autoinhibitory mechanism exists whereby the C2 domain binds the HECT domain and inhibits its catalytic activity^9–11^. Given that only a small segment of the C2 domain is present in Nedd4-1(NE), it is uncertain whether this autoinhibitory mechanism is preserved. To address this, we utilized a HECT domain mutation (Y604A) that perturbs C2 domain binding, thus blocking the autoinhibitory mechanism and consequently favoring the active state of the HECT domain^20^. Our results show that unlike Nedd4-1, in Nedd4-1(NE), the analogous mutation (Y1023A) does not enhance catalytic activity, presumably due to the lack of a preserved autoinhibitory mechanism (Fig. 2C). Therefore, unlike the C2 domain of Nedd4-1, the NE region of Nedd4-1(NE) does not negatively regulate the HECT domain, a factor which likely contributes to the enhanced catalytic activity of Nedd4-1(NE) compared to Nedd4-1.

### Nedd4-1(NE) binds 14-3-3 proteins

**Figure 3:**
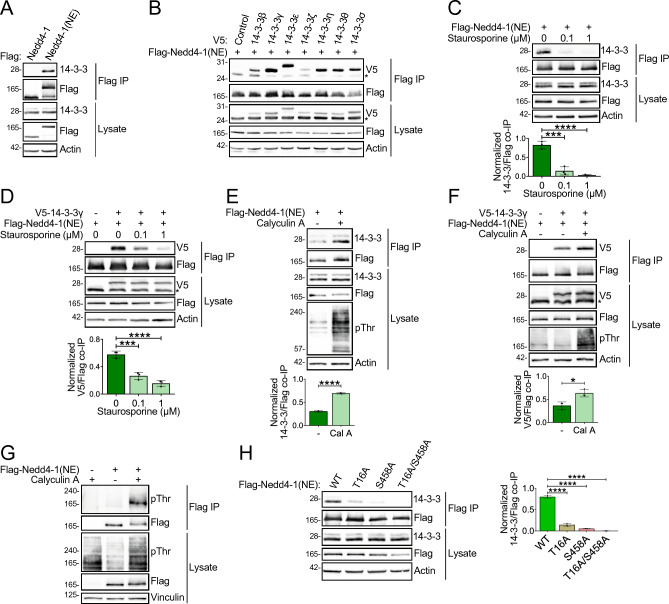
Nedd4-1(NE) binds 14-3-3 in a phosphorylation-dependent manner. (**A**,**H**) HEK293T cells were transfected with the indicated Flag-tagged constructs. Cell lysates were subjected to Flag IP and the indicated proteins were detected by immunoblotting. (**B**–**G**) 293 T-REx cells expressing 3xFlag-Nedd4-1(NE) following tetracycline induction were (**B,D,F**) transfected with V5-tagged 14-3-3 constructs, (**C**,**D**) treated with staurosporine (2 h at the indicated dose), and/or (**E**,**F**) treated with Calyculin A (15 min; 50 nM). Cell lysates were treated as in (**A**). Asterisks (*) indicate non-specific bands. Data are means ± S.D. of 3 independent experiments. Statistical significance was determined using (**C**,**D**,**H**) one-way ANOVA, or (**E**,**F**) Student’s t test (*p < 0.05; ***p < 0.001; ****p < 0.0001).

**Figure 4:**
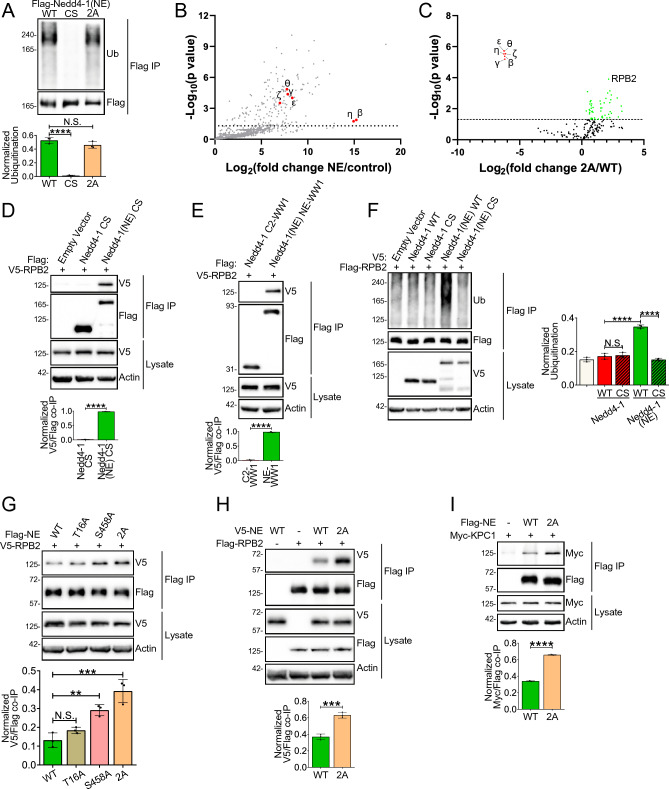
14-3-3 binding site mutations in the NE region promote substrate binding. (**A**,**F**) HEK293T cells were transfected with the indicated constructs, with the Nedd4-1(NE) 2A mutant representing the T16A/S458A double mutant. Cell lysates were subjected to Flag IP following addition of 1% SDS and boiling to dissociate protein complexes. Ubiquitination was detected by immunoblotting for ubiquitin (Ub). (**B**,**C**) HEK293T cells were transfected with Flag-tagged NE region (WT or 2A mutant) or an empty vector control. Cell lysates were subjected to Flag IP and interacting proteins were identified by mass spectrometry. In (**B**), data points above the dashed line (p > 0.05) represent significant interactors of the WT NE region, with 14-3-3 isoforms highlighted in red. In (**C**), data points highlighted in red represent proteins that are significantly decreased in the 2A compared to the WT data set, while data points highlighted in green represent proteins that are significantly increased in the 2A compared to the WT data set. (**D**,**E**,**G**–**I**) HEK293T cells were transfected with the indicated constructs. Cell lysates were subjected to Flag IP and the indicated proteins were detected by immunoblotting. All data are means ± S.D. of 3 independent experiments. Statistical significance was determined using (**A**,**F**,**G**) one-way ANOVA, or (**D**,**E**,**H**,**I**) Student’s t test (**p < 0.01; ***p < 0.001; ****p < 0.0001; *N.S.* not significant).

To help decipher the function of the NE region, we searched for its binding partners. To identify these partners, we performed affinity purification of Flag-tagged constructs including full-length Nedd4-1(NE), the NE region alone, and Nedd4-1, followed by mass spectrometry (MS). From this proteomic analysis, we searched for proteins that interacted with Nedd4-1(NE) and its NE region, but not with Nedd4-1. Among the top proteins that satisfied these criteria were 6 members of the 14-3-3 family of proteins (Table S1; see also Fig. 4B). In mammals, this family consists of seven structurally similar isoforms (β, γ, ε, ζ, η, θ, σ) involved in diverse regulatory functions^21^. We confirmed the interaction between Nedd4-1(NE) and both endogenous 14-3-3 (Fig. 3A) and all seven overexpressed 14-3-3 isoforms (Fig. 3B). Importantly, canonical Nedd4-1 was incapable of binding 14-3-3 (Fig. 3A), suggesting an isoform-specific interaction mediated by the NE region.

### 14-3-3 binding to Nedd4-1(NE) depends on two key N-terminal phosphoresidues

14-3-3 proteins contain a well-conserved ligand-binding pocket^22,23^, a region capable of recognizing specific motifs centered on phosphoserine (pSer) or phosphothreonine (pThr) residues^24–26^. Given the importance of phosphorylation in mediating 14-3-3 binding, we investigated whether we could enhance or perturb the interaction between Nedd4-1(NE) and 14-3-3 by inhibiting cellular kinases or phosphatases. Our results show that the interaction between Nedd4-1(NE) and either endogenous 14-3-3 proteins (Fig. 3C) or overexpressed 14-3-3γ (Fig. 3D) was inhibited by treatment with the general kinase inhibitor staurosporine, while treatment of cells with Calyculin A, a potent inhibitor of PP1 and PP2A phosphatases, enhanced this interaction (Fig. 3E,F). Consistent with this observation, we found detectable levels of Nedd4-1(NE) phosphorylation in cells treated with Calyculin A (Fig. 3G).

To identify the phosphosites on Nedd4-1(NE) required for 14-3-3 binding, we used 14-3-3-Pred, a 14-3-3-binding phosphopeptide predictor^27^ (Table S2). We created serine/threonine to alanine mutants of the highest scoring predicted phosphosites and evaluated their ability to bind 14-3-3 by co-IP. Our results show that mutating Thr^16^ or Ser^458^ to alanine diminished the interaction between Nedd4-1(NE) and 14-3-3, while mutation of both residues (T16A/S458A; “2A”) completely abolished it (Fig. 3H). Collectively, these results demonstrate that the interaction between Nedd4-1(NE) and 14-3-3 is dependent on phosphorylation of two residues, Thr^16^ and Ser^458^, both unique to the NE region of Nedd4-1(NE).

### 14-3-3 proteins regulate substrate binding to Nedd4-1(NE)

Members of the 14-3-3 family interact with a wide variety of proteins and consequently regulate various functions of target proteins, including catalytic activity, subcellular localization, and protein–protein interactions^21^. To determine how 14-3-3 binding may regulate Nedd4-1(NE) function, we compared wild-type (WT) Nedd4-1(NE) with the 2A mutant that cannot bind 14-3-3. We found that these mutations did not alter the catalytic activity of Nedd4-1(NE) (Fig. 4A), nor did they alter its subcellular localization (not shown).

To explore the possibility that the interactome of Nedd4-1(NE) is altered by 14-3-3 binding, we performed quantitative IP-MS, comparing the interactomes of the WT and the 2A NE regions of Nedd4-1(NE) in HEK293T cells (Fig. 4B,C). Consistent with our earlier experiments described in Fig. 3 and Table S1, we identified six members of the 14-3-3 family as significant interactors of the WT NE region (Fig. 4B). Of all the interacting proteins that were significantly different between the WT and 2A samples, these six proteins were the only ones to exhibit reduced binding to the 2A mutant (Fig. 4C and Table S3). On the other hand, 50 proteins were identified with significantly enhanced binding to the 2A mutant than the WT NE region (Fig. 4C and Table S3), suggesting that these mutations stabilize or enhance various protein–protein interactions mediated by the NE region. To validate this hypothesis, we focused on the RPB2 subunit of RNA polymerase II, the most significant hit in the aforementioned cohort. Indeed, our results show that RPB2 bound Nedd4-1(NE), but not Nedd4-1 (Fig. 4D,E). In accord, we show that RPB2 is a substrate of Nedd4-1(NE), but not Nedd4-1 (Fig. 4F). Consistent with the IP-MS data, we found that RPB2 interacts with the 2A mutant of the NE region to a greater extent than the WT NE region (Fig. 4G,H). Moreover, we found that KPC1, an independently identified binding partner and substrate of Nedd4-1(NE), also exhibits increased binding to the 2A mutant compared to the WT NE region (Fig. 4I). Taken together, these data demonstrate that mutations in Thr^16^ and Ser^458^ have a global effect on stabilizing or enhancing substrate binding to the NE region of Nedd4-1(NE) by preventing either phosphorylation or 14-3-3 binding.

## Discussion

Our studies here analyzed Nedd4-1(NE), a poorly characterized isoform of the ubiquitin ligase Nedd4-1, which is widely distributed in tissues and cells (although expressed at lower levels than the canonical Nedd4-1 under steady-state conditions). Specifically, our work distinguishes Nedd4-1(NE) from the canonical Nedd4-1 isoform with regards to its catalytic activity, substrates, and mechanisms of regulation. Our work represents the first direct comparison of alternatively spliced isoforms of Nedd4-1.

While several modes of regulation of catalytic activity of Nedd4-1 have been described (e.g.^28,29^), the most pertinent to our studies here are those that implicate the C2 domain, which is largely absent in Nedd4-1(NE). Residues absent in Nedd4-1(NE) comprise calcium-binding sites, HECT domain-binding sites, and Src kinase phosphorylation sites, all of which are involved in regulating autoinhibition of enzymatic activity of Nedd4-1^10,30^. Accordingly, we found that Nedd4-1(NE) is more active than Nedd4-1, likely due to the absence of the autoinhibitory mechanism brought about by C2:HECT domains interaction.

We identified 14-3-3 proteins as unique and prominent interactors of the NE region of Nedd4-1(NE). Importantly, our results indicate that 14-3-3 cannot bind canonical Nedd4-1, providing evidence for the first time of different binding partners for these two alternatively spliced isoforms. 14-3-3 proteins were previously reported to regulate the closely related Nedd4-1 relative, Nedd4-2 (also known as Nedd4-like/Nedd4L)^31–33^. Specifically, 14-3-3 binds Nedd4-2 at pSer and pThr sites, blocking its binding to the epithelial sodium channel ENaC, thus suppressing Nedd4-2-dependent ubiquitination and degradation of ENaC. Moreover, recent biophysical/crystallography analysis of the Nedd4-2:14-3-3 interaction revealed that 14-3-3 proteins prevent intramolecular interactions between Nedd4-2 WW3/WW4 domains and the HECT domain, potentially affecting catalytic activity in addition to regulating binding to ENaC^34,35^.

Our quantitative IP-MS data not only identified novel binding partners of Nedd4-1(NE), which are not dependent on binding to its WW domains, but also identified binding partners and substrates that preferentially bind to the unphosphorylated NE region. Our results indicate that 14-3-3 binding exerts a more widespread effect on Nedd4-1(NE) than Nedd4-2 by regulating universal substrate binding, including binding to seemingly unrelated substrates such as RPB2 and KPC1. While we found that the mutation of 14-3-3 binding phosphosites in the NE region enhanced substrate binding, we do not know whether that is directly caused by the absence of 14-3-3 binding, or by the loss of phosphorylation at these residues. If the former is true, phosphorylation may recruit 14-3-3, thus blocking substrate binding, while if the latter is true, 14-3-3 binding may simply protect the phosphorylation of these residues, thereby limiting substrate binding.

Our above quantitative IP-MS experiments identified the RPB2 subunit of RNA polymerase II as an isoform-specific binding partner and substrate of Nedd4-1(NE). Previous reports had identified the canonical Nedd4-1, as well as its yeast ortholog Rsp5, as the ubiquitin ligase responsible for ubiquitination of RNA polymerase II^36,37^. However, these reports focused on the RPB1 subunit of the complex, while our proteomic screen identified the RPB2 subunit as a binding partner of Nedd4-1(NE). Thus, we propose that the different subunits of RNA polymerase II are ubiquitinated by different isoforms of Nedd4-1, with RPB1 targeted by the canonical Nedd4-1 through a WW domain-mediated interaction, and RPB2 targeted by Nedd4-1(NE) through a NE region-mediated interaction.

In summary, our study characterized the ubiquitin ligase Nedd4-1(NE), differentiating it from the canonical Nedd4-1 isoform with regards to its catalytic activity and binding partners. Moreover, we identified an isoform-specific mechanism by which substrate binding is regulated.

## Methods

### Cell culture and transfection

HEK293T (ATCC #CRL-1573) and 293 T-REx (Thermo Fisher Scientific #R78007) cells were cultured in Dulbecco's Modified Eagle Medium (DMEM), supplemented with 10% fetal bovine serum and antibiotic–antimycotic. 293 T-REx stable cell lines were generated by recombinase-mediated DNA recombination using the Flp-In system (Thermo Fisher Scientific) and maintained in DMEM containing 100 µg/ml hygromycin B. Recombinant protein expression was induced by treating 293 T-REx cells with 1 µg/ml tetracycline for 24 h. Transfections were performed using PolyJet (SignaGen) or PolyEZ (MoCellutions) reagents.

### Constructs

The DNA fragment encoding the NE region of Nedd4-1(NE) was generated by PCR using primers (F: 5′-gcagccagaagaagcaatacttac-3′; R: 5′-cttgcaagaattagacagttcatttgtgc-3′), with HeLa cell-derived cDNA serving as a template. cDNA encoding the NE region or the full-length Nedd4-1(NE) were synthesized and/or subcloned into mammalian expression vectors using Gibson assembly, Gateway cloning, and restriction digestion and ligation. Catalytically inactive (CS) or 14-3-3 binding-deficient (2A) Nedd4-1(NE) mutants were generated via site-directed mutagenesis. 14-3-3β, 14-3-3γ, 14-3-3ε, 14-3-3θ, 14-3-3σ, RPB2, and KPC1 plasmids were obtained from the human ORFeome^38^ (distributed by SPARC Drug Discovery, The Hospital for Sick Children, Toronto, ON, Canada). 14-3-3ζ (#48798) and 14-3-3η (#116887) plasmids were from Addgene.

### cDNA preparation and PCR

Total RNA was extracted from cells using the PureLink RNA Mini Kit (Thermo Fisher Scientific) and reverse transcribed to cDNA using the High-Capacity cDNA Reverse Transcription Kit (Thermo Fisher Scientific). The human tissue cDNA panel was purchased from Zyagen. Nedd4-1(NE) expression was assessed by PCR using primers that can recognize multiple alternatively spliced isoforms of Nedd4-1(NE). Amplicon identity was confirmed by Sanger sequencing. RT-qPCR analysis was performed using Power SYBR Green (Thermo Fisher Scientific) following the manufacturer’s instructions. Primers used are listed in Table S4.

### Immunoprecipitation (IP), ubiquitination assays, and immunoblotting

Cells were lysed in lysis buffer (50 mM HEPES pH 7.5, 150 mM NaCl, 1% Triton X-100, 10% glycerol, 1.5 mM MgCl_2_, 1 mM EGTA) freshly supplemented with 1 mM PMSF, 10 µg/ml leupeptin, 10 µg/ml aprotinin, and 10 µg/ml pepstatin. Flag IPs were performed by incubating 1 mg clarified cell lysate with anti-Flag M2 affinity gel (Sigma-Aldrich) for 2 h at 4 °C. For ubiquitination assays, lysates were boiled in 1% sodium dodecyl sulfate (SDS) for 5 min at 95 °C prior to IP. Proteins from cell lysates and/or IPs were resolved by SDS-PAGE and immunoblotted with the indicated antibodies. Antibodies used in this study are listed in Table S5. Immunoblots were imaged using a ChemiDoc MP imaging system (Bio-Rad) and analyzed using Image Lab software (Bio-Rad).

### Affinity purification–mass spectrometry

HEK293T cells were transfected with Flag-tagged constructs. Cells were lysed and Flag IPs were performed using 10 mg of clarified cell lysate. Subsequent sample preparation, data collection, and data searches were carried out at the SPARC BioCentre at The Hospital for Sick Children (Toronto, ON, Canada). Proteins were reduced using 10 mM DTT (60 min, 60 °C), alkylated using 20 mM iodoacetamide (45 min, RT), and digested overnight at 37 °C using 2 µg trypsin (Pierce). Peptides were lyophilized using a SpeedVac centrifuge, desalted using C18 ZipTips (Millipore), and resuspended in 2% acetonitrile and 0.1% formic acid. Samples were analyzed by liquid chromatography tandem mass spectrometry (LC–MS/MS) using an EASY-nLC 1200 nano-LC system coupled to an Orbitrap Fusion Lumos Tribrid mass spectrometer (Thermo Fisher Scientific). Peptides were eluted over 60 min at 250 nl/min with a 0 to 80% acetonitrile gradient in 0.1% formic acid. MS1 spectra were measured with a resolution of 120,000, an automatic gain control (AGC) target of 400,000, a maximum ion injection time (MIIT) of 50 ms, and a scan range from 375 to 1500 *m/z*. MS2 scans were performed using an ion trap with an AGC target of 10,000, a MIIT of 10 ms, and a scan range from 200 to 1400 *m/z*. The raw MS data files were searched against the UniProt database (Uniprot_UP000005640, downloaded 15/09/2020) restricted to *Homo sapiens* using Proteome Discoverer version 2.5.0.400 (Thermo Fisher Scientific). Data were analyzed using Scaffold version 5.1.2. Quantitative values (normalized total precursor intensity) of all proteins were calculated for each sample in three independent experiments. Values below the detection threshold of 10^6^ were assigned a value of 10^6^. Fold change was calculated between the mean quantitative values and significance was assessed using the Student’s t test.

### Supplementary Information

Supplementary Information.

## Data Availability

Available in the Supplementary Material and also upon request to drotin@sickkids.ca.

## References

[1] Hershko A, Ciechanover A (1998). The ubiquitin system. Annu. Rev. Biochem..

[2] Rotin D, Kumar S (2009). Physiological functions of the HECT family of ubiquitin ligases. Nat. Rev. Mol. Cell Biol..

[3] Cao XR (2008). Nedd4 controls animal growth by regulating IGF-1 signaling. Sci. Signal.

[4] Lin Q (2010). HECT E3 ubiquitin ligase Nedd4-1 ubiquitinates ACK and regulates epidermal growth factor (EGF)-induced degradation of EGF receptor and ACK. Mol. Cell. Biol..

[5] Persaud A (2011). Nedd4-1 binds and ubiquitylates activated FGFR1 to control its endocytosis and function. EMBO J..

[6] Sicari D, Weber J, Maspero E, Polo S (2022). The NEDD4 ubiquitin E3 ligase: A snapshot view of its functional activity and regulation. Biochem. Soc. Trans..

[7] Bernassola F, Chillemi G, Melino G (2019). HECT-type E3 ubiquitin ligases in cancer. Trends Biochem. Sci..

[8] Plant PJ, Yeger H, Staub O, Howard P, Rotin D (1997). The C2 domain of the ubiquitin protein ligase Nedd4 mediates Ca2+-dependent plasma membrane localization. J. Biol. Chem..

[9] Wang J (2010). Calcium activates Nedd4 E3 ubiquitin ligases by releasing the C2 domain-mediated auto-inhibition. J. Biol. Chem..

[10] Persaud A (2014). Tyrosine phosphorylation of NEDD4 activates its ubiquitin ligase activity. Sci. Signal.

[11] Wiesner S (2007). Autoinhibition of the HECT-type ubiquitin ligase Smurf2 through its C2 domain. Cell.

[12] Staub O (1996). WW domains of Nedd4 bind to the proline-rich PY motifs in the epithelial Na+ channel deleted in Liddle's syndrome. EMBO J..

[13] Kanelis V, Rotin D, Forman-Kay JD (2001). Solution structure of a Nedd4 WW domain-ENaC peptide complex. Nat. Struct. Biol..

[14] Huibregtse JM, Scheffner M, Beaudenon S, Howley PM (1995). A family of proteins structurally and functionally related to the E6-AP ubiquitin-protein ligase. Proc. Natl. Acad. Sci. U. S. A..

[15] Dunn DM (2002). Common variant of human NEDD4L activates a cryptic splice site to form a frameshifted transcript. J. Hum. Genet..

[16] Flasza M, Gorman P, Roylance R, Canfield AE, Baron M (2002). Alternative splicing determines the domain structure of WWP1, a Nedd4 family protein. Biochem. Biophys. Res. Commun..

[17] Soond SM, Chantry A (2011). Selective targeting of activating and inhibitory Smads by distinct WWP2 ubiquitin ligase isoforms differentially modulates TGFbeta signalling and EMT. Oncogene.

[18] Strausberg RL (2002). Generation and initial analysis of more than 15,000 full-length human and mouse cDNA sequences. Proc. Natl. Acad. Sci. U. S. A..

[19] Persaud A (2009). Comparison of substrate specificity of the ubiquitin ligases Nedd4 and Nedd4-2 using proteome arrays. Mol. Syst. Biol..

[20] Mari S (2014). Structural and functional framework for the autoinhibition of Nedd4-family ubiquitin ligases. Structure.

[21] Bridges D, Moorhead GB (2005). 14-3-3 proteins: A number of functions for a numbered protein. Sci. STKE.

[22] Liu D (1995). Crystal structure of the zeta isoform of the 14-3-3 protein. Nature.

[23] Xiao B (1995). Structure of a 14-3-3 protein and implications for coordination of multiple signalling pathways. Nature.

[24] Muslin AJ, Tanner JW, Allen PM, Shaw AS (1996). Interaction of 14-3-3 with signaling proteins is mediated by the recognition of phosphoserine. Cell.

[25] Yaffe MB (1997). The structural basis for 14-3-3:phosphopeptide binding specificity. Cell.

[26] Brunet A (1999). Akt promotes cell survival by phosphorylating and inhibiting a Forkhead transcription factor. Cell.

[27] Madeira F (2015). 14-3-3-Pred: Improved methods to predict 14-3-3-binding phosphopeptides. Bioinformatics.

[28] Attali I (2017). Ubiquitylation-dependent oligomerization regulates activity of Nedd4 ligases. EMBO J..

[29] Jiang H, Thomas SN, Chen Z, Chiang CY, Cole PA (2019). Comparative analysis of the catalytic regulation of NEDD4-1 and WWP2 ubiquitin ligases. J. Biol. Chem..

[30] Escobedo A (2014). Structural basis of the activation and degradation mechanisms of the E3 ubiquitin ligase Nedd4L. Structure.

[31] Ichimura T (2005). 14-3-3 proteins modulate the expression of epithelial Na+ channels by phosphorylation-dependent interaction with Nedd4-2 ubiquitin ligase. J. Biol. Chem..

[32] Bhalla V (2005). Serum- and glucocorticoid-regulated kinase 1 regulates ubiquitin ligase neural precursor cell-expressed, developmentally down-regulated protein 4–2 by inducing interaction with 14-3-3. Mol. Endocrinol..

[33] Chandran S (2011). Neural precursor cell-expressed developmentally down-regulated protein 4–2 (Nedd4-2) regulation by 14-3-3 protein binding at canonical serum and glucocorticoid kinase 1 (SGK1) phosphorylation sites. J. Biol. Chem..

[34] Joshi R (2022). Nedd4-2 binding to 14-3-3 modulates the accessibility of its catalytic site and WW domains. Biophys. J..

[35] Pohl P, Joshi R, Petrvalska O, Obsil T, Obsilova V (2021). 14-3-3-protein regulates Nedd4-2 by modulating interactions between HECT and WW domains. Commun. Biol..

[36] Anindya R, Aygün O, Svejstrup JQ (2007). Damage-induced ubiquitylation of human RNA polymerase II by the ubiquitin ligase Nedd4, but not Cockayne syndrome proteins or BRCA1. Mol. Cell.

[37] Huibregtse JM, Yang JC, Beaudenon SL (1997). The large subunit of RNA polymerase II is a substrate of the Rsp5 ubiquitin-protein ligase. Proc. Natl. Acad. Sci. U. S. A..

[38] The ORFeome Collaboration (2016). The ORFeome Collaboration: A genome-scale human ORF-clone resource. Nat. Methods.

