# Supplementary material for: Primate-specific isoform of Nedd4-1 regulates substrate binding via Ser/Thr phosphorylation and 14-3-3 binding
Source: Sci Rep. 2023 Oct 20;13:17903. doi: 10.1038/s41598-023-44761-9 (PMC10589272; doi:10.1038/s41598-023-44761-9)

Supplementary Material

Primate-specific isoform of Nedd4-1 regulates substrate binding via Ser/Thr phosphorylation and 14-3-3 binding

George Kefalas[1,2] and Daniela Rotin[1,2,*]

[1]Cell Biology Program, the Hospital for Sick Children, and [2]Biochemistry Department, University of Toronto, Toronto, Ontario, Canada, M5G 0A4

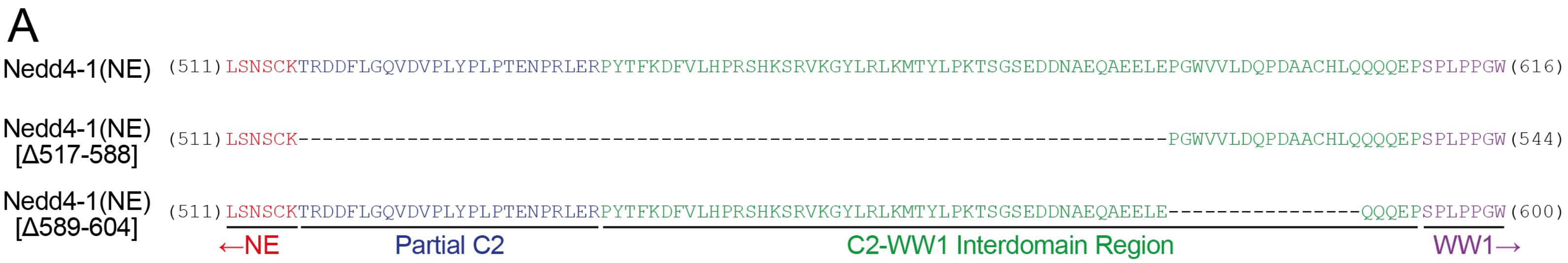

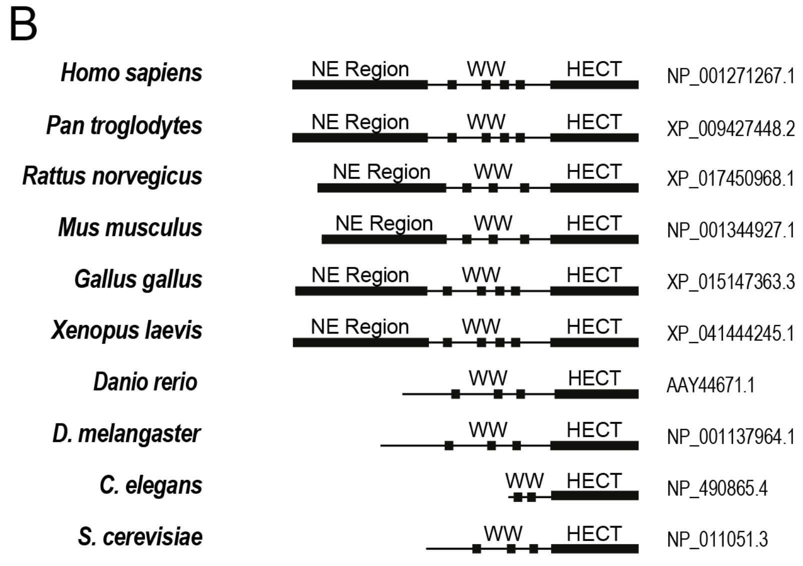

% amino acid identity vs. human	
NE Region	HECT
99%	100%
62%	97%
62%	97%
48%	94%
41%	90%
N/A	88%
	73%
	65%
	58%

Figure S1: Nedd4-1(NE) isoforms in humans and conservation of the NE region in alternatively spliced isoforms of Nedd4 throughout evolution

(**A**) Protein sequences highlighting the differences in amino acids between the isoforms of human Nedd4-1(NE). The longest isoform of Nedd4-1(NE), used in our studies, contains a 516-amino acid NE region and a partial C2 domain. Nedd4-1(NE) Δ517-588 does not have a partial C2 domain and is missing part of the C2-WW1 interdomain region proximal to the NE region. Nedd4-1(NE) Δ589-604 is missing part of the C2-WW1 interdomain region proximal to the WW1 domain. (**B**) Schematic representation of Nedd4 isoforms formed by alternative splicing in different species. Protein accession numbers (NCBI) are listed on the right of each isoform. Homologous isoforms of human Nedd4-1(NE) were identified by searching species-specific protein databases. NE regions were identified in Nedd4 homologs in *Pan troglodytes* (chimpanzee), *Rattus norvegicus* (rat), *Mus musculus* (mouse), *Gallus gallus* (chicken), and *Xenopus laevis* (frog). No homologous isoforms containing NE regions were identified in *Danio rerio* (zebrafish), *Drosophila melanogaster* (fruit fly), *Caenorhabditis elegans* (worm), or *Saccharomyces cerevisiae* (yeast). All homologs are named Nedd4 (or Nedd4-1), with the exception of *C. elegans* (HECT-type E3 ubiquitin transferase) and *S. cerevisiae* (Rsp5). Table on the right indicates the % amino acid identity shared between human Nedd4-1(NE) and the homologous isoform of each species with respect to their NE regions and HECT domains.

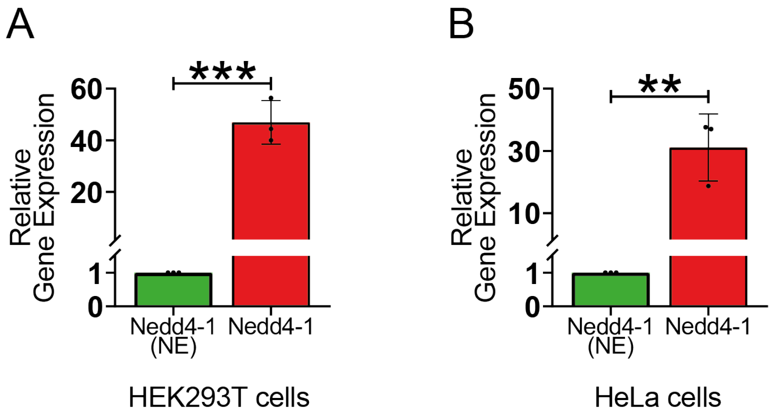

Figure S2: Nedd4-1 isoform-specific mRNA expression

mRNA expression of Nedd4-1(NE) (using a NE-targeting primer) and Nedd4-1 (using a C2-targeting primer) was assessed by RT-qPCR using cDNA derived from (**A**) HEK293T cells or (**B**) HeLa cells. Data are means ± S.D. of 3 independent experiments. Statistical significance was determined using Student's t test (**: $p < 0.01$; ***: $p < 0.001$).

	Negative control (Empty vector)	NE region	Nedd4-1(NE)	Nedd4-1
**14-3-3β**	n = 0/2	n = 2/2<br>pc = 14-41%	n = 2/2<br>pc = 27-41%	n = 0/2
**14-3-3γ**	n = 0/2	n = 2/2<br>pc = 14-32%	n = 2/2<br>pc = 14-28%	n = 0/2
**14-3-3ε**	n = 0/2	n = 2/2<br>pc = 18-65%	n = 2/2<br>pc = 24-56%	n = 0/2
**14-3-3ζ**	n = 0/2	n = 2/2<br>pc = 27-41%	n = 2/2<br>pc = 27-44%	n = 0/2
**14-3-3η**	n = 0/2	n = 1/2<br>pc = 33%	n = 1/2<br>pc = 33%	n = 0/2
**14-3-3θ**	n = 0/2	n = 2/2<br>pc = 10-36%	n = 2/2<br>pc = 22-36%	n = 0/2
**14-3-3σ**	n = 0/2	n = 1/2<br>pc = 15%	n = 0/2	n = 0/2

Table S1: proteomic analysis identifies 14-3-3 proteins as interactors of Nedd4-1(NE)
HEK293T cells were transfected with the indicated Flag-tagged constructs, and cell lysates were subjected to Flag IP. Interacting proteins were identified by mass spectrometry. This table summarizes the peptide coverage of the 14-3-3 proteins identified in each IP-MS sample (n: biological replicates; pc: peptide coverage across the biological replicates). Zero peptide fragments corresponding to 14-3-3 proteins were identified in the control or Nedd4-1 IP-MS samples.

Site	Peptide	ANN	PSSM	SVM	Consensus
458	ILRRSI**S**LGGA	0.975	1.488	2.297	1.586
16	AARRSN**T**YPLS	0.96	1.722	1.577	1.42
265	PLKRYS**S**LVIF	0.817	1.02	0.954	0.93
232	IFSRST**S**TDPF	0.766	0.999	1.02	0.928
193	SDRSSY**T**FPPS	0.694	0.793	0.521	0.669
274	IFPRSP**S**TTRP	0.699	0.79	0.375	0.621
387	TQRKAA**T**LDCE	0.821	0.485	0.541	0.615
444	ELERPH**S**QMNK	0.659	0.903	0.27	0.611
349	PLHRKG**S**LQKK	0.612	0.826	0.324	0.587
276	PRSPST**T**RPTS	0.677	0.709	0.347	0.578
43	RPTRIS**T**SNVV	0.512	0.803	0.046	0.454
331	GLRLSK**T**ICTP	0.644	0.409	0.215	0.423
279	PSTTRP**T**SPTS	0.583	0.408	-0.064	0.309
410	IKLNSD**S**EYIK	0.58	0.201	0.105	0.295
142	NDGHLV**S**SPAI	0.479	0.378	-0.023	0.278
233	FSRSTS**T**DPFV	0.499	0.595	-0.371	0.241
221	SVPNRN**T**TPCE	0.451	0.535	-0.274	0.237
222	VPNRNT**T**PCEI	0.419	0.441	-0.167	0.231
508	DSNRDC**T**NELS	0.492	0.668	-0.497	0.221
215	DSTSNR**S**VPNR	0.535	0.388	-0.275	0.216
44	PTRIST**S**NVVQ	0.515	0.388	-0.293	0.203
264	IPLKRY**S**SLVI	0.468	0.225	-0.094	0.2
490	LLIKFA**S**GNEG	0.549	0.154	-0.183	0.173
22	TYPLSE**T**SGDD	0.452	0.188	-0.363	0.092
73	QSQERS**S**VPSS	0.264	0.44	-0.455	0.083
176	GSCASI**T**SGGS	0.453	0.076	-0.297	0.078
157	LSNFST**S**DNGS	0.366	0.121	-0.309	0.059
514	TNELSN**S**CK--	0.327	0.289	-0.449	0.056
304	QFIISP**S**EIAH	0.255	0.195	-0.305	0.049
280	STTRPT**S**PTSL	0.21	0.374	-0.509	0.025

Table S2: predicted 14-3-3 binding sites in the NE region

Thr (T) and Ser (S) residues in the NE region were scored for their predicted ability to bind 14-3-3 proteins using 14-3-3-Pred[27]. Total score is a consensus of three algorithms: artificial neural network (ANN), position-specific scoring matrix (PSSM), and support vector machines (SVM).

Gene/Protein	Log₂ Normalized Total Precursor Intensity									log₂ fold change (2A/WT)	-log₁₀ p value
	EV-1	EV-2	EV-3	2A-1	2A-2	2A-3	WT-1	WT-2	WT-3		
**YWHAE (14-3-3ε)**	**27.91**	**28.06**	**29.53**	**30.41**	**30.72**	**30.54**	**37.00**	**36.89**	**36.60**	**-6.27**	**5.73**
**YWHAQ (14-3-3θ)**	**27.29**	**27.43**	**28.18**	**28.99**	**29.17**	**29.25**	**35.58**	**35.41**	**35.15**	**-6.25**	**5.72**
**YWHAZ (14-3-3ζ)**	**29.46**	**27.56**	**29.10**	**29.47**	**29.72**	**29.50**	**35.85**	**35.83**	**35.42**	**-6.14**	**5.53**
**YWHAH (14-3-3η)**	**16.61**	**16.61**	**28.17**	**29.13**	**29.07**	**29.16**	**35.68**	**35.36**	**35.12**	**-6.27**	**5.53**
**YWHAB (14-3-3β)**	**27.40**	**16.61**	**16.61**	**29.01**	**29.32**	**29.36**	**35.68**	**35.41**	**35.20**	**-6.20**	**5.38**
**YWHAG (14-3-3γ)**	**27.29**	**27.37**	**28.45**	**29.09**	**29.47**	**29.45**	**35.81**	**35.54**	**35.26**	**-6.20**	**5.19**
**POLR2B (RPB2)**	**16.61**	**16.61**	**16.61**	**28.07**	**28.41**	**28.24**	**26.23**	**25.86**	**26.15**	**2.16**	**3.91**
SELENOF	16.61	16.61	16.61	24.64	24.00	24.43	22.07	21.86	22.36	2.26	3.15
FAM83H	16.61	16.61	16.61	27.15	27.18	27.22	26.72	26.64	26.80	0.47	3.10
FAT3	16.61	16.61	16.61	29.56	29.67	28.90	27.34	27.28	27.25	2.09	3.01
POLE	16.61	16.61	16.61	26.13	26.31	26.09	24.35	23.69	23.36	2.38	2.88
FAT4	16.61	16.61	16.61	25.08	26.16	25.88	22.39	23.17	23.00	2.86	2.69
HSPBP1	16.61	16.61	16.61	25.73	25.19	24.99	23.09	23.32	23.67	1.94	2.66
CELSR2	16.61	16.61	16.61	29.15	29.17	28.52	26.16	26.55	27.12	2.34	2.58
HSPA4L	21.52	16.61	25.02	30.39	30.59	30.72	29.76	29.95	29.79	0.73	2.52
AIFM1	26.38	25.46	26.92	33.05	32.89	32.52	31.67	31.09	31.25	1.48	2.50
TUBB	33.51	32.11	33.76	37.26	37.27	36.96	36.42	36.55	36.39	0.71	2.49
TUBB2B	33.06	31.71	33.26	36.72	36.84	36.52	35.99	36.12	35.92	0.68	2.45
FAT1	16.61	16.61	16.61	29.57	29.66	29.87	27.30	26.88	28.14	2.26	2.39
UGGT1	16.61	16.61	16.61	26.60	27.56	27.45	25.45	25.43	25.33	1.80	2.38
BBS7	16.61	16.61	16.61	25.60	25.37	25.66	24.37	23.54	24.19	1.51	2.31
HSPA8	33.35	32.09	33.42	36.76	36.57	36.83	36.04	35.69	36.05	0.79	2.31
TUBB4B	33.50	31.81	33.42	37.06	37.11	36.76	36.33	36.40	36.25	0.65	2.30
FLNA	31.99	30.99	32.47	34.45	34.84	34.85	33.84	33.93	34.08	0.76	2.19
LRP4	16.61	16.61	16.61	26.84	27.75	27.04	24.26	22.98	24.86	3.18	2.17
FLNC	16.61	28.09	16.61	34.30	34.32	33.98	33.59	33.12	33.14	0.91	2.08
AMBRA1	16.61	16.61	16.61	24.71	25.12	24.84	23.35	23.78	22.69	1.62	2.05
PRAME	16.61	16.61	16.61	23.49	23.60	23.36	21.33	22.21	22.32	1.53	2.05
AKAP11	26.45	24.16	25.60	28.37	28.36	28.10	27.43	27.33	27.78	0.76	2.03
TUBB8	16.61	16.61	16.61	35.74	35.75	35.38	34.97	35.14	34.95	0.60	1.95
ZWINT	16.61	16.61	16.61	24.43	24.65	24.57	23.66	23.91	24.13	0.65	1.88
PRKDC	30.59	28.01	30.88	32.68	32.83	32.85	32.37	32.36	32.56	0.36	1.86
FREM2	16.61	16.61	16.61	29.35	29.65	28.65	28.12	27.46	27.45	1.54	1.85
CELSR1	16.61	16.61	16.61	27.69	28.05	29.92	24.60	25.67	25.71	3.23	1.84
DNAJA1	27.54	27.23	28.33	31.50	31.79	31.11	30.55	30.63	30.72	0.83	1.84
STUB1	16.61	16.61	16.61	29.22	29.26	29.70	28.08	26.21	27.04	2.28	1.81
SCO2	16.61	16.61	23.47	28.27	27.92	27.78	27.03	27.04	27.46	0.82	1.81
MCCC1	16.61	16.61	16.61	23.95	23.23	23.50	22.83	22.51	22.60	0.91	1.77
HSPA4	26.67	25.58	27.34	31.05	31.11	30.76	30.57	30.29	30.16	0.63	1.76
DCAF7	16.61	16.61	16.61	27.08	28.26	27.38	24.79	24.53	26.23	2.39	1.70
HSPA5	31.20	30.02	30.90	34.73	34.60	34.73	34.38	33.83	33.98	0.62	1.66
TRIM32	16.61	16.61	16.61	24.47	25.62	25.63	22.17	23.33	23.73	2.16	1.63
SEC23A	16.61	16.61	21.52	25.32	25.03	24.26	22.12	23.35	23.53	1.87	1.58
PPP6R1	21.11	16.61	16.61	25.13	25.07	25.44	24.04	22.59	24.06	1.65	1.52
PCDH7	16.61	16.61	16.61	26.71	29.66	27.35	25.13	24.47	25.28	2.94	1.47
POLR1B	16.61	16.61	21.53	25.98	26.05	24.95	23.33	24.24	24.57	1.62	1.46
TARS2	16.61	16.61	16.61	25.09	26.23	25.90	23.79	24.43	22.23	2.26	1.43
ISYNA1	16.61	16.61	16.61	25.12	24.70	24.69	24.22	22.66	22.62	1.67	1.42
N4BP2	16.61	16.61	16.61	24.63	25.45	24.99	22.56	22.20	24.23	2.03	1.41
SPC24	16.61	16.61	16.61	25.36	25.06	25.29	24.86	23.82	23.92	1.03	1.40
SDF4	16.61	16.61	16.61	28.31	28.44	28.04	27.80	27.05	27.65	0.76	1.39
SPTLC1	16.61	16.61	23.49	26.39	26.81	26.05	25.20	25.79	25.73	0.85	1.37
EML4	16.61	16.61	16.61	22.31	22.52	23.17	21.82	21.98	21.93	0.75	1.35
BRCA2	21.55	16.61	16.61	27.25	27.19	27.35	26.86	25.41	25.49	1.34	1.33
HSPA1B	33.73	32.20	32.87	37.04	36.72	36.53	36.44	35.92	35.71	0.74	1.32
FANCI	22.73	16.61	23.21	28.49	29.27	28.15	27.90	27.55	27.14	1.10	1.30

Table S3: IP-MS significant hits in the WT NE region vs. 2A NE region

HEK293T cells were transfected with the indicated Flag-tagged constructs, and cell lysates were subjected to Flag IP. Interacting proteins were identified by mass spectrometry. Quantitative values (normalized total precursor intensity) are listed for three independent experiments for each sample (EV: empty vector; 2A: T16A/S458A double mutant in the NE region; WT: wild-type NE region). Values below the detection threshold of 10^6 were assigned a value of 10^6. This table includes all proteins that significantly differed ($p > 0.05$) between the 2A and WT samples and is sorted based on statistical significance. Proteins of interest in this manuscript are highlighted in bold.

Primer	Use	Sequence (5' → 3')
Nedd4-1(NE) Forward	Detection of Nedd4-1(NE) isoforms by PCR, generating amplicons of different sizes [Figures 1B-D]	GGGGGACCATCTCAGTTACTC
Nedd4-1(NE) Reverse		CGGAAGACTCTCGGTTGTCA
NE Forward	Detection of all isoforms of Nedd4-1(NE) by RT-qPCR [Figure S2]	TCCAGCCTTAAGCACAATCG
NE Reverse		GTGGAACATCCACTTGACCT
C2 Forward	Detection only of canonical Nedd4-1 by RT-qPCR [Figure S2]	GTGGAGGTGTTCGGGCTC
C2 Reverse		GCAAGGCCTATTCCGGCTAT

Table S4: primers used in this study

Antibody	Source	Product #
14-3-3 (pan), rabbit	Cell Signaling Technology	8312
β-Actin, mouse	Sigma-Aldrich	A2228
Flag M2, mouse	Sigma-Aldrich	F1804
Flag, rabbit	Cell Signaling Technology	2368
Myc, mouse	Sigma-Aldrich	05-724
Phosphothreonine, mouse	Cell Signaling Technology	9686
Ubiquitin, mouse	Santa Cruz	sc-8017
V5, mouse	Bio-Rad	MCA1360
V5, rabbit	Cell Signaling Technology	13202
Vinculin, mouse	Santa Cruz	sc-25336
Anti-mouse IgG, HRP-linked	Cell Signaling Technology	7076
Anti-rabbit IgG, HRP-linked	Cell Signaling Technology	7074

Table S5: antibodies used in this study and their sources

Original Blots

Figure 1B

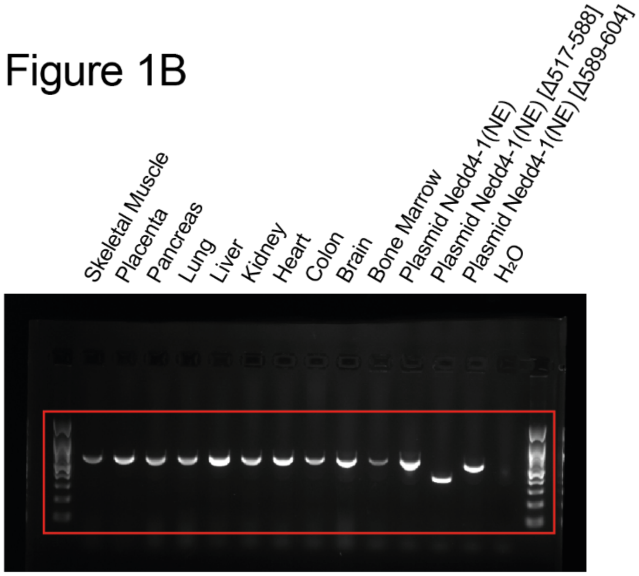

Figure 1C

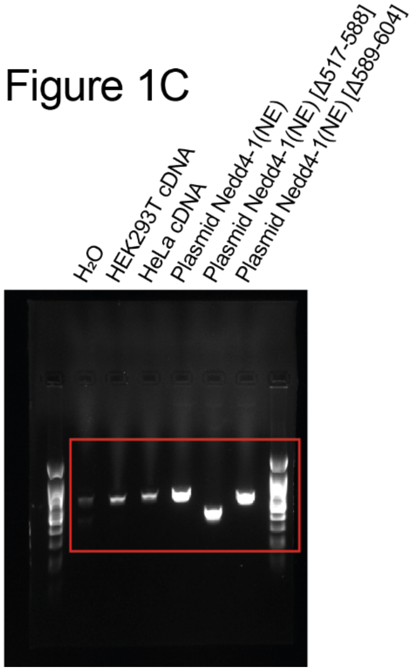

Figure 1D

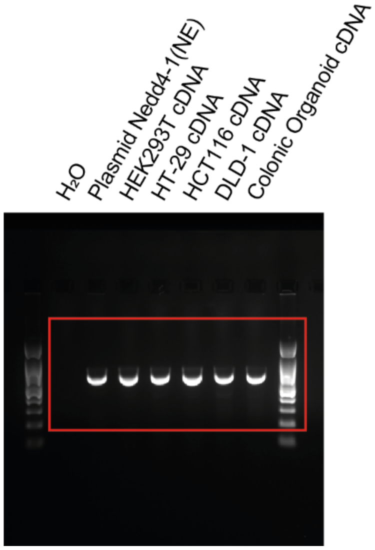

Figure 2A

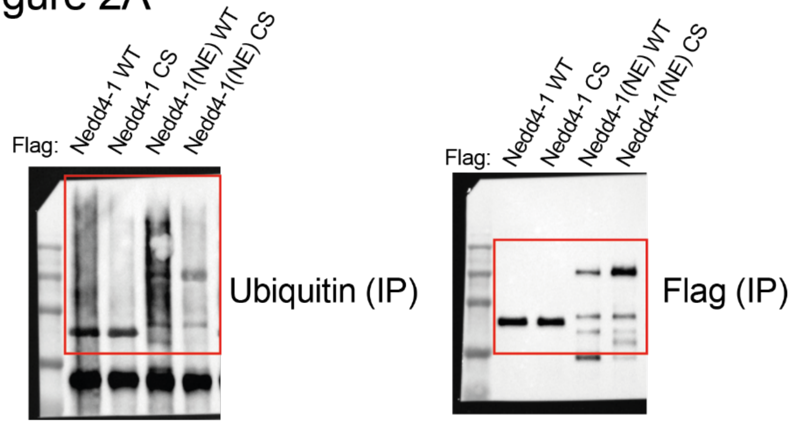

Figure 2B

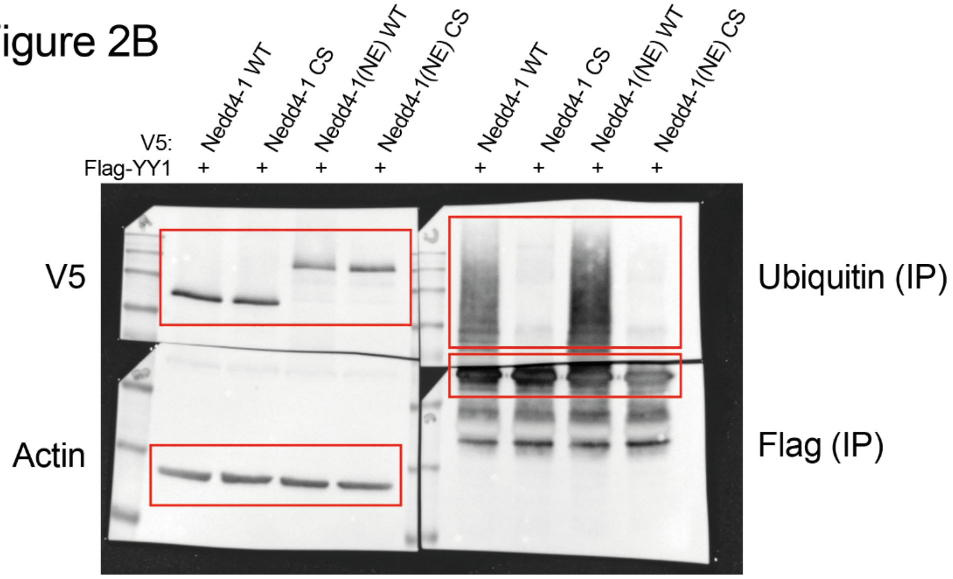

Figure 2C

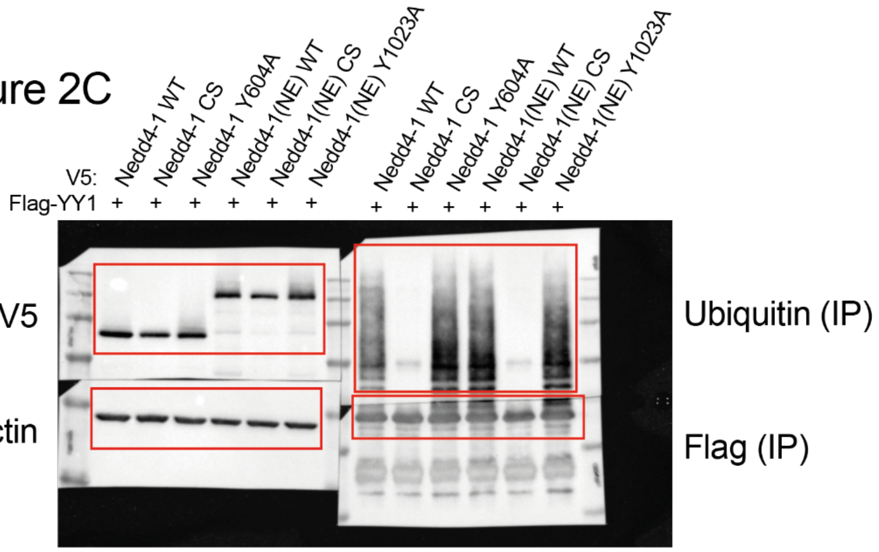

Figure 3A

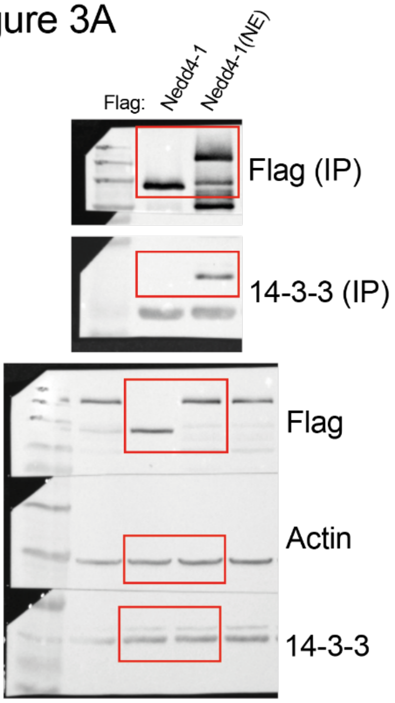

Figure 3B

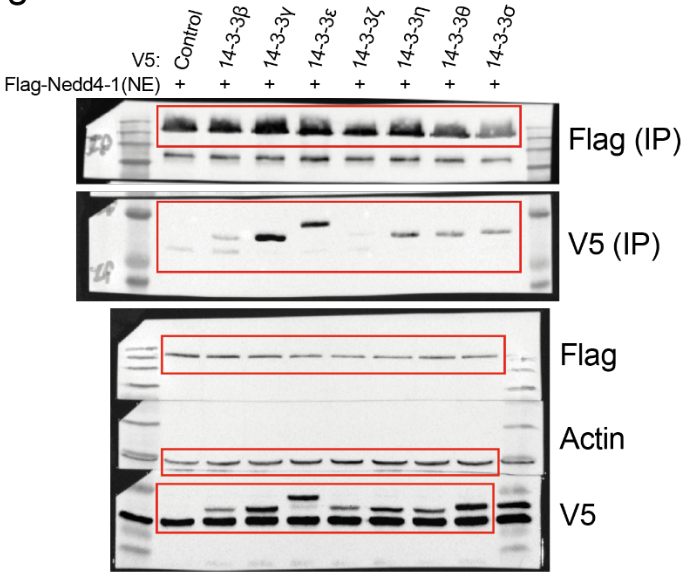

Figure 3C

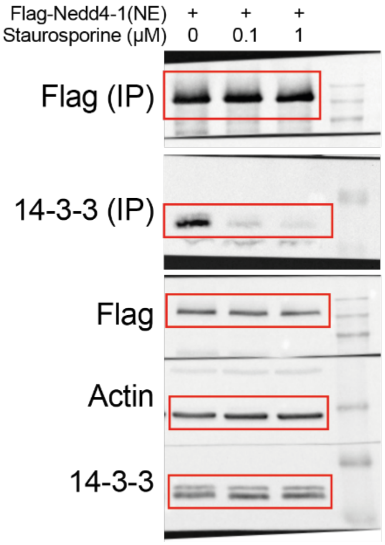

Figure 3D

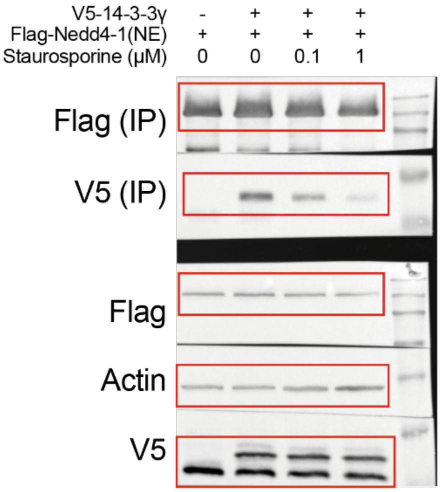

Figure 3E

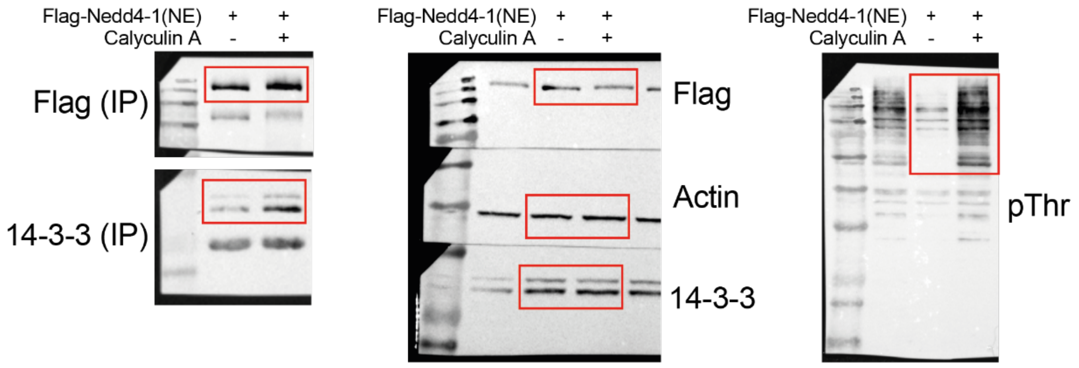

Figure 3F

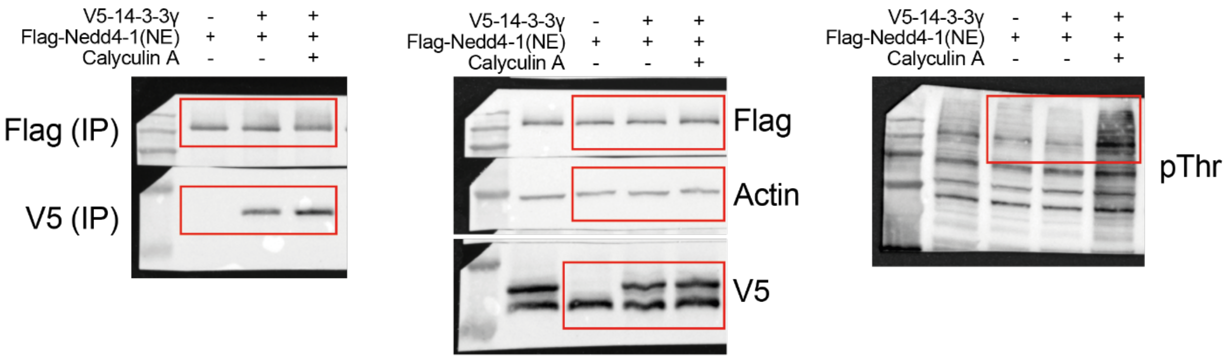

Figure 3G

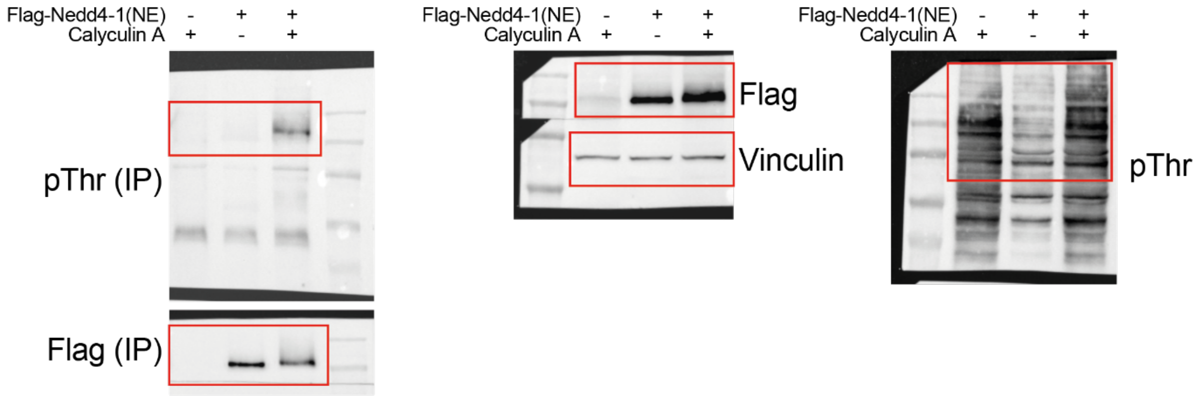

Figure 3H

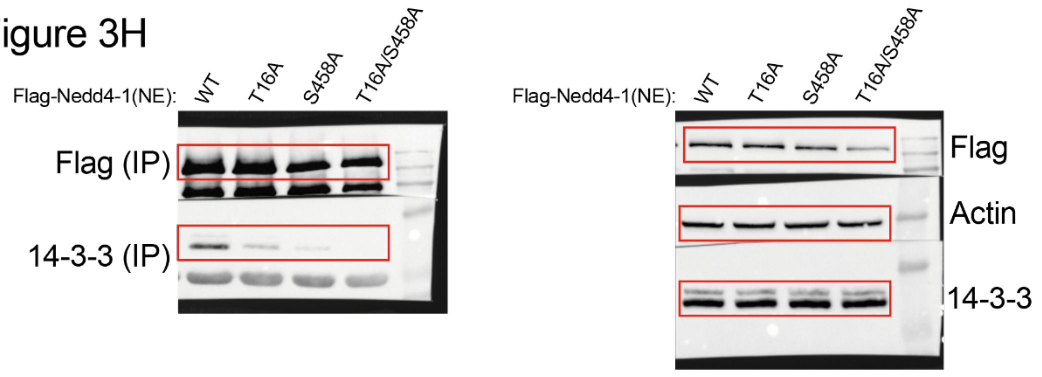

Figure 4A

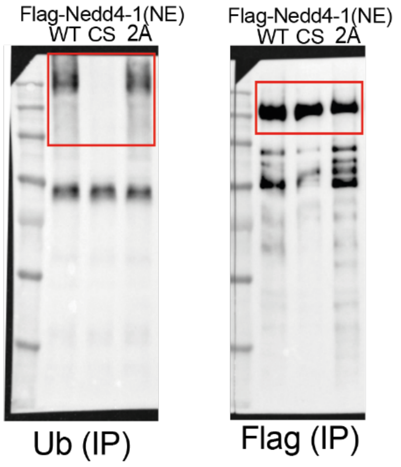

Figure 4D

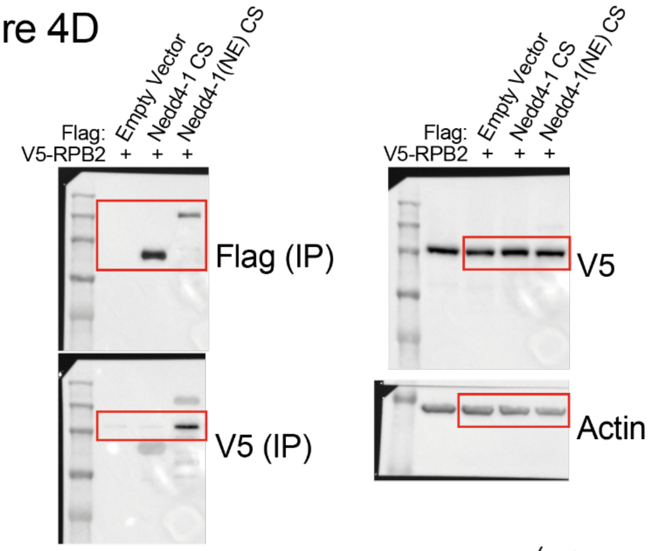

Figure 4E

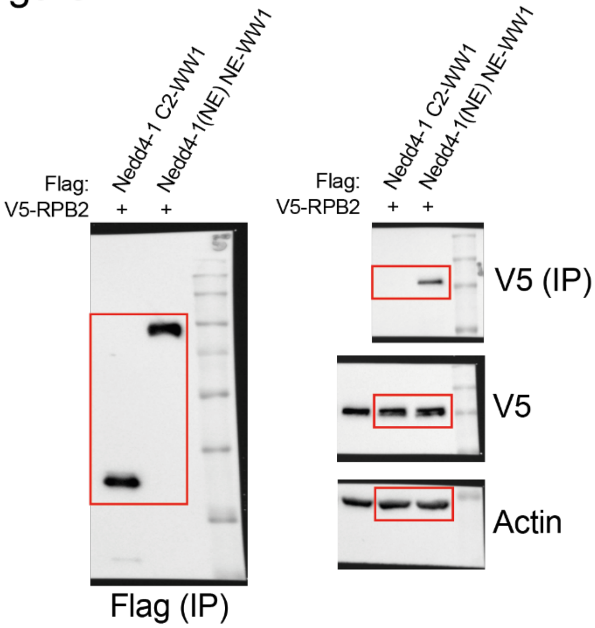

Figure 4F

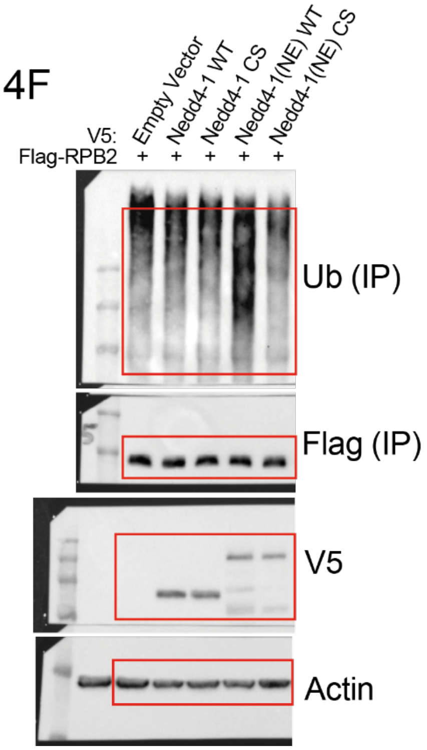

Figure 4G

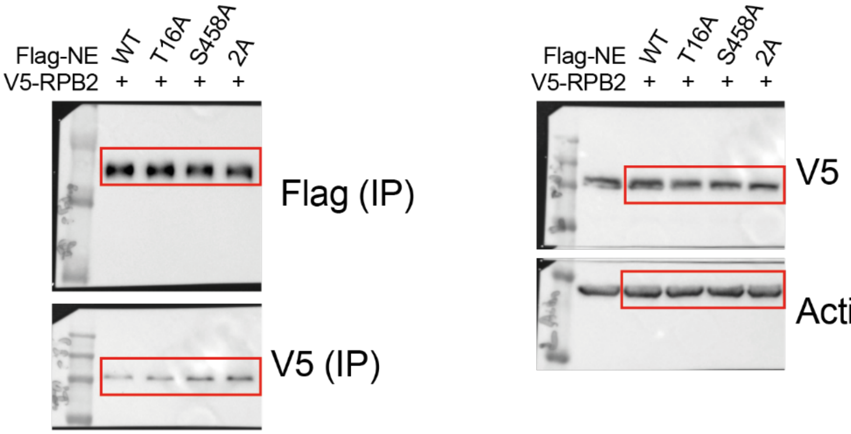

Figure 4H

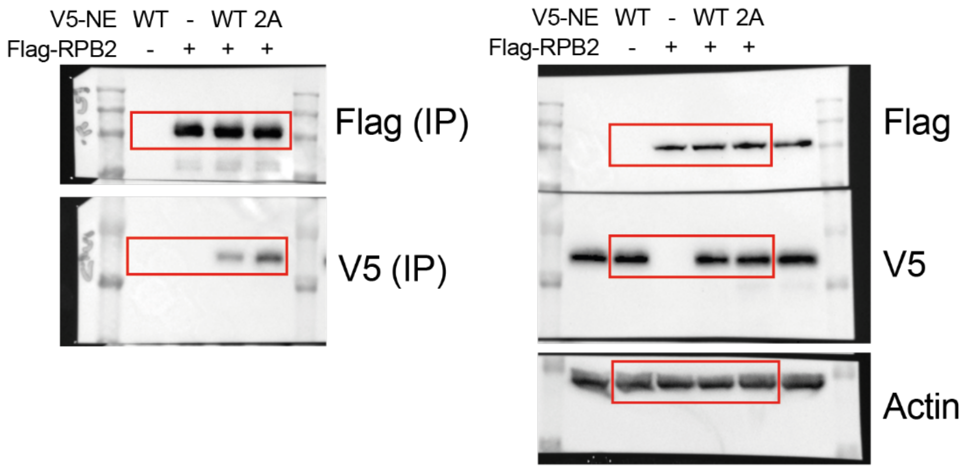

Figure 4I

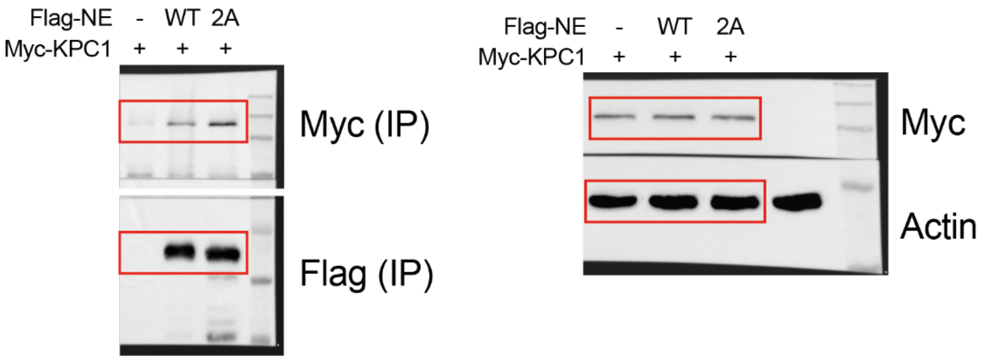

Supplement: Supplementary file 1 — Supplementary Information. [file 41598_2023_44761_MOESM1_ESM.pdf]
